# *GNP2* Encodes a High-Specificity Proline Permease in Candida albicans

**DOI:** 10.1128/mbio.03142-21

**Published:** 2022-01-25

**Authors:** Enrico Garbe, Pedro Miramón, Franziska Gerwien, Nico Ueberschaar, Louisa Hansske-Braun, Philipp Brandt, Bettina Böttcher, Michael Lorenz, Slavena Vylkova

**Affiliations:** a Septomics Research Center, Friedrich Schiller University, Jena, Germany; b Leibniz Institute for Natural Product Research and Infection Biology, Hans Knöll Institute, Jena, Germany; c Department of Microbiology and Molecular Genetics, McGovern Medical School, University of Texas Health Science Center at Houston, Houston, Texas, USA; d Mass Spectrometry Platform, Friedrich Schiller University, Jena, Germany; Geisel School of Medicine at Dartmouth

**Keywords:** *Candida albicans*, amino acid transport, proline, filamentation, oxidative stress

## Abstract

The tight association of Candida albicans with the human host has driven the evolution of mechanisms that permit metabolic flexibility. Amino acids, present in a free or peptide-bound form, are abundant carbon and nitrogen sources in many host niches. In C. albicans, the capacity to utilize certain amino acids, like proline, is directly connected to fungal morphogenesis and virulence. Yet the precise nature of proline sensing and uptake in this pathogenic fungus has not been investigated. Since C. albicans encodes 10 putative orthologs of the four Saccharomyces cerevisiae proline transporters, we tested deletion strains of the respective genes and identified Gnp2 (CR_09920W) as the main C. albicans proline permease. In addition, we found that this specialization of Gnp2 was reflected in its transcriptional regulation and further assigned distinct substrate specificities for the other orthologs, indicating functional differences of the C. albicans amino acid permeases compared to the model yeast. The physiological relevance of proline uptake is exemplified by the findings that strains lacking *GNP2* were unable to filament in response to extracellular proline and had a reduced capacity to damage macrophages and impaired survival following phagocytosis. Furthermore, *GNP2* deletion rendered the cells more sensitive to oxidative stress, illustrating new connections between amino acid uptake and stress adaptation in C. albicans.

## INTRODUCTION

Candida albicans is a common member of the human microbiota colonizing mucosal surfaces such as the oral cavity, the vagina, and the gastrointestinal tract (1, 2). Despite a close and commensal relationship, predisposing conditions such as prolonged antibiotic treatment, immunosuppression, extended hospitalization, and disruption of anatomical barriers render the host susceptible to infections by C. albicans. Clinical manifestations of candidiasis range from mucosal diseases (e.g., oral, esophageal, and vulvovaginal) to life-threatening deep-seated infections that can affect virtually any organ (3, 4). C. albicans owns a variety of fitness and virulence factors that account for its success as a commensal and a pathogen, like robust stress responses, polymorphism, rapid adhesion to host tissues, and a cytolytic peptide toxin (5–7).

Metabolic plasticity, the ability to utilize a wide array of nutrients and quickly adapt to environmental changes, is another key adaptation to the host (8–11). The nutritional compositions of the myriad host niches that C. albicans inhabits differ significantly, and simple sugars are often limited in these environments (9, 12–14). Thus, alternative nutrient sources like lipids, organic acids, amino sugars, and amino acids are exploited by C. albicans to support growth and satisfy its energy needs. Many of these are abundant and readily accessible within the host, and mutants unable to utilize these compounds are often attenuated in virulence (13, 15–19).

Most host tissues are rich in accessible amino acids and peptides, and C. albicans possesses multiple utilization mechanisms such as secreted proteases, oligopeptide transporters, and amino acid permeases (AAPs), exceeding the repertoire of nonpathogenic fungi (19–21). The genes required for the uptake of these nutrients are often induced under virulence-associated conditions, like upon confrontation with macrophages or neutrophils (22, 23). Amino acids serve not only as ready-made building blocks for protein biosynthesis but also as valuable nitrogen and carbon sources, avidly metabolized for energy production and gluconeogenesis. Besides their nutritional value, amino acids directly affect fungal virulence in multiple ways, for instance, through the induction of filamentation (24). Arginine and proline are particularly effective in this process (25–28). Both amino acids are converted to α-ketoglutarate via the mitochondrial proline catabolism pathway, fueling the tricarboxylic acid (TCA) cycle (28). Subsequently, oxidative phosphorylation generates ATP, the accumulation of which induces morphogenesis by the activation of the Ras1/cAMP/protein kinase A (PKA) pathway (28, 29). This metabolic pathway was shown to be directly connected to fungal survival upon macrophage phagocytosis, as mutants defective in proline catabolism were impaired in intraphagosomal filamentation and macrophage survival (28).

In C. albicans, amino acid uptake is mediated by at least 24 AAPs, with only a few being functionally characterized. In this fungus, functional annotations often rely on predictions from the respective orthologs in the well-studied model yeast Saccharomyces cerevisiae (24, 30–32). A key component in the regulation of AAP expression is the conserved SPS (Ssy1-Ptr3-Ssy5) system. Abundant extracellular amino acids, like arginine, are sensed by Ssy1, which serves as a trigger for the proteolytic activation of the transcription factor Stp2, resulting in its nuclear shuttling and the expression of multiple AAP genes (33, 34). The functionality of this pathway is crucial for fungal growth on multiple amino acids and linked to pathogenicity, as *stp2*Δ or SPS component mutants are attenuated *in vivo* (16, 35, 36). Interestingly, extracellular proline does not induce the SPS system in C. albicans (28). Furthermore, the components contributing to the uptake of this amino acid have not been investigated. In S. cerevisiae, four permeases contribute to proline transport: ScAgp1 and ScGnp1, regulated by the SPS system, and ScGap1 and ScPut4, subject to NCR (nitrogen catabolite repression) (37). However, in C. albicans, proline utilization is also NCR independent, suggesting that proline uptake is mediated differently in this fungus compared to the model yeast (38).

In this work, we identified Gnp2 (CR_09920W) as the main C. albicans proline permease, important for growth and filamentation in response to proline *in vitro*. Despite the presence of additional orthologs of the S. cerevisiae proline permeases, they do not contribute to proline uptake. Furthermore, *gnp2*Δ strains have an impaired capacity to damage macrophages despite the ability to filament following phagocytosis. Finally, the deletion of *GNP2* renders the cells sensitive to oxidative stress, pointing toward an important role of proline uptake in metabolic and stress adaptation within the host.

## RESULTS

### C. albicans encodes multiple orthologs of the S. cerevisiae proline permeases.

**FIG 1:**
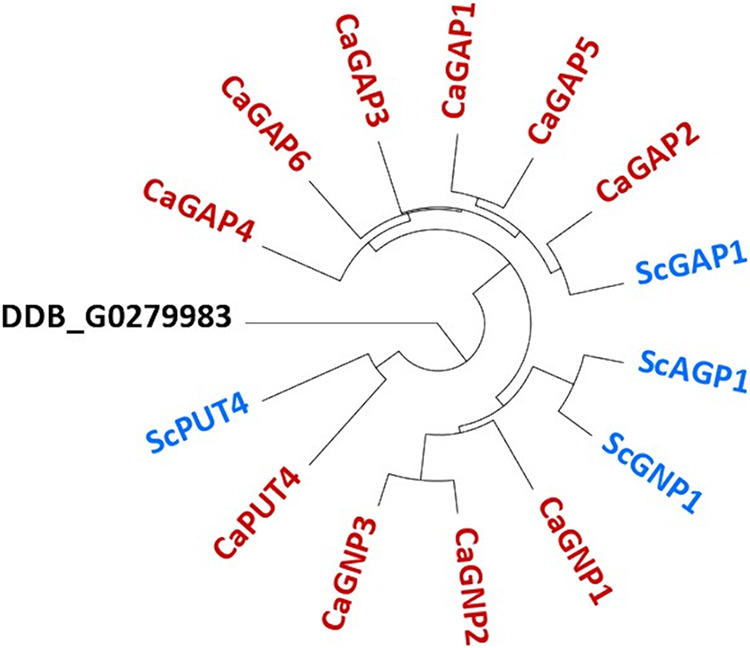
Phylogenetic tree of S. cerevisiae proline permeases and their C. albicans orthologs. The phylogenetic tree was assembled using protein sequences with Geneious Prime (v2020.2.3) (tree builder, unweighted pair group method using average linkages [UPGMA], and default settings). The Dictyostelium discoideum hypothetical protein DDB_G0279983 (GenBank accession number XP_641447) was used as the outgroup. S. cerevisiae AAPs are indicated in blue, and C. albicans AAPs are in red.

In order to identify the C. albicans proline permease(s), we first examined the genome for orthologs of the four confirmed proline permeases in S. cerevisiae: ScAgp1, ScGnp1, ScGap1, and ScPut4. C. albicans encodes 10 orthologs, which cluster into three subgroups (Gnp1 to -3, Gap1 to -6, and Put4) (Fig. 1; see also Table S1A in the supplemental material). In general, BLASTp analysis showed that both S. cerevisiae and C. albicans AAPs are highly similar on the protein level within the same species, making precise assignments difficult (Table S1B). Nevertheless, we either generated or utilized available single-deletion strains or strains carrying multiple AAP knockouts from the respective subgroups. The resulting set was then used to comprehensively characterize the substrate specificity of the deleted permeases.

10.1128/mBio.03142-21.8 TABLE S1 Putative proline permeases in C. albicans. Download Table S1, XLSX file, 0.03 MB. Copyright © 2022 Garbe et al. 2022 Garbe et al. https://creativecommons.org/licenses/by/4.0/ This content is distributed under the terms of the Creative Commons Attribution 4.0 International license.

### Characterization of C. albicans amino acid permease substrate specificities.

To investigate amino acid specificity, we applied a screening approach using sterically similar toxic amino acid analogs. An AAP deletion strain displaying resistance to a toxic amino acid analog would indicate that the respective permease is required for the uptake of the sterically similar amino acid.

**FIG 2:**
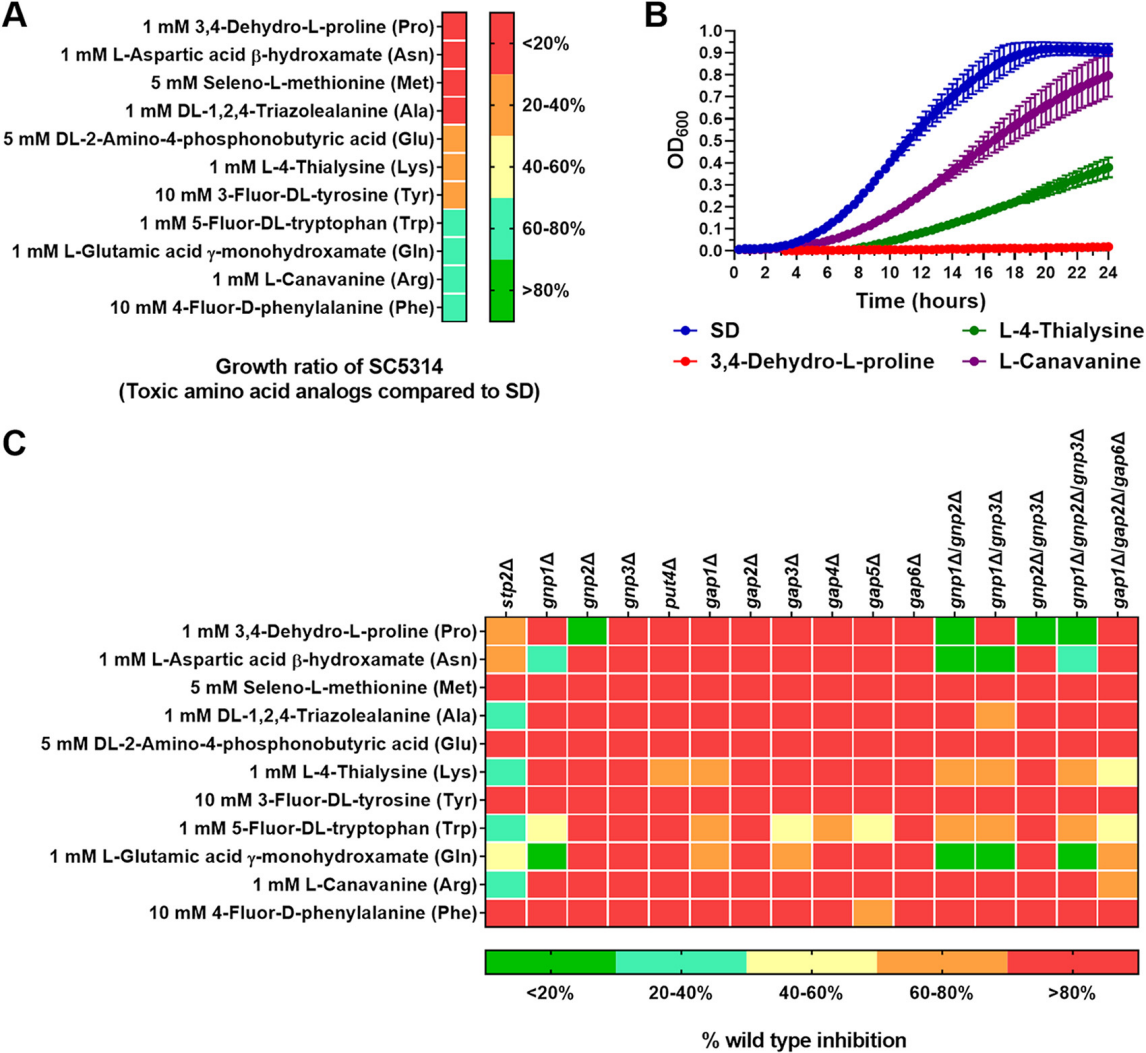
Screen of C. albicans AAP mutants for resistance to toxic amino acid analogs reveals novel phenotypes. (A) Relative growth of the SC5314 wild type in SD minimal medium supplemented with toxic amino acid analogs compared to growth in SD medium alone. The analogous proteinogenic amino acid is indicated in parentheses. (B) Representative growth curves for SC5314 in SD medium alone and supplemented with 3,4-dehydro-l-proline, l-canavanine, or l-4-thialysine. (C) Growth inhibition of AAP deletion strains and the *stp2*Δ mutant relative to the wild type on each toxic amino acid analog. Red indicates growth inhibition of the AAP deletion strain equivalent to that of the wild type, while green indicates unaffected growth of the AAP deletion strain equal to the growth in SD medium alone.

At first, we tested amino acid analogs shown to impair microbial growth, as well as other commercially available analogs, for their capacity to inhibit the growth of the SC5314 wild-type strain when added to amino-acid-free SD minimal medium. Using this approach, we identified toxic analogs of 11 amino acids: Pro, Asn, Gln, Glu, Arg, Lys, Ala, Phe, Tyr, Met, and Trp (Fig. 2A and B; Table S2A). Next, the single AAP deletion strains and the *stp2*Δ strain, impaired in the uptake of multiple amino acids, were tested with the established screening method in order to identify AAP substrate specificities and the requirement of Stp2 for their utilization (Fig. 2C). For better comparability, the growth of each deletion strain in the presence of the particular analogs was expressed as a percentage of the respective inhibition of the wild type (see Materials and Methods; Table S2B). The retained growth of the *stp2*Δ strain on toxic analogs of Stp2-dependent amino acids confirmed the functionality of this approach.

10.1128/mBio.03142-21.9 TABLE S2 Toxic amino acid analogs/growth rates of C. albicans. Download Table S2, XLSX file, 0.4 MB. Copyright © 2022 Garbe et al. 2022 Garbe et al. https://creativecommons.org/licenses/by/4.0/ This content is distributed under the terms of the Creative Commons Attribution 4.0 International license.

The most striking phenotype was observed for the *gnp2*Δ strain, which was completely resistant to the proline analog 3,4-dehydro-l-proline but otherwise sensitive to all other tested analogs (Fig. 2C). Resistance to the proline analog was observed in all strains deleted for *GNP2*, namely, *gnp1*Δ/*gnp2*Δ, *gnp2*Δ/*gnp3*Δ, and *gnp1*Δ/*gnp2*Δ/*gnp3*Δ. Since no other AAP deletion resulted in resistance to 3,4-dehydro-l-proline, we inferred that Gnp2 is critical for C. albicans proline uptake. Consequently, the reintegration of *GNP2* into the *gnp2*Δ strain resulted in restored sensitivity to 3,4-dehydro-l-proline (Fig. S1). Of note, the *stp2*Δ strain displayed moderate resistance to the proline analog, suggesting that the deletion of this transcription factor influences proline utilization.

10.1128/mBio.03142-21.1 FIG S1 Complementation for *GNP2* restores sensitivity to 3,4-dehydro-l-proline. Growth of the indicated C. albicans strains in SD medium and SD medium supplemented with 1 mM 3,4-dehydro-l-proline was assessed in a 96-well microtiter plate (final volume of 100 μL) at 20-min intervals for 24 h. The mean of all replicates is displayed (biological duplicates and technical triplicates). Download FIG S1, TIF file, 0.2 MB. Copyright © 2022 Garbe et al. 2022 Garbe et al. https://creativecommons.org/licenses/by/4.0/ This content is distributed under the terms of the Creative Commons Attribution 4.0 International license.

Aside from the prominent role of Gnp2 in proline uptake, we were able to assign putative functional roles to other AAPs in C. albicans. For instance, strains carrying *GNP1* deletions displayed high-level resistance to the glutamine and asparagine analogs and intermediate resistance to the tryptophan analog (Fig. 2C). The retained capacity of the *gnp1*Δ strain to grow in the presence of the asparagine analog was unique among all tested strains, suggestive of specialized uptake by Gnp1. Additional permeases are likely involved in glutamine and tryptophan uptake: the *gap1*Δ, *gap3*Δ, and *gap1*Δ/*gap2*Δ/*gap6*Δ strains were slightly resistant to the glutamine analog, while *gap1*Δ, *gap4*Δ, and, to a lesser extent, *gap3*Δ, *gap5*Δ, and *gap1*Δ/*gap2*Δ/*gap6*Δ strains were resistant to the tryptophan analog 5-fluor-dl-tryptophan. Furthermore, strains carrying deletions of *PUT4*, *GAP1*, and *GNP1*/*2*/*3* exhibited slight to moderate resistance to the lysine analog l-4-thialysine (Fig. 2C).

To expand the results from the toxic amino acid analog screen, we tested the *GNP* deletion strains for growth on SD medium supplemented with single amino acids as the sole nitrogen source. We also included the *stp2*Δ strain as its growth characteristics under the tested conditions are well defined (33, 39). Overall, the amino acid capacity to promote fungal growth varied greatly; e.g., glutamine and asparagine promoted robust growth, whereas tyrosine or lysine was the least capable of supporting C. albicans growth as a nitrogen source (Fig. S2A). The *stp2*Δ strain was impaired in growth on multiple nitrogen sources but not on proline (Fig. S2B), which is well in line with previous findings (33). Strains in which *GNP2* was deleted consistently had impaired growth on proline as the sole nitrogen source (Fig. S2B). Furthermore, the *gnp1*Δ/*gnp2*Δ strain showed moderately impaired growth on methionine, while the *gnp1*Δ strain grew less well on threonine, and no impairment was visible on asparagine or glutamine (Fig. S2B). Oddly, strains lacking *GNP1* or *GNP3* had markedly increased (up to 260%) growth on lysine as a nitrogen source compared to SC5314 (Table S2C).

10.1128/mBio.03142-21.2 FIG S2 C. albicans Gnp2 is essential for growth on proline as either a carbon or a nitrogen source. (A) The SC5314 wild type was cultivated in liquid SD-based minimal medium with the indicated amino acids as the sole nitrogen source. Growth rates were calculated relative to growth in SD medium with ammonium sulfate as the sole nitrogen source (100%). (B) Growth of AAP deletion strains and the *stp2*Δ mutant on different nitrogen sources relative to SC5314 growth (100%). (C) The indicated C. albicans strains were grown in liquid medium containing proline as the sole carbon source (YNB, ammonium sulfate if indicated, and 10 mM proline [pH 4.5]). Growth was assessed in a 96-well microtiter plate at 20-min intervals for 72 h. The mean of all replicates is displayed (biological duplicates and technical triplicates). Download FIG S2, TIF file, 0.8 MB. Copyright © 2022 Garbe et al. 2022 Garbe et al. https://creativecommons.org/licenses/by/4.0/ This content is distributed under the terms of the Creative Commons Attribution 4.0 International license.

**FIG 3:**
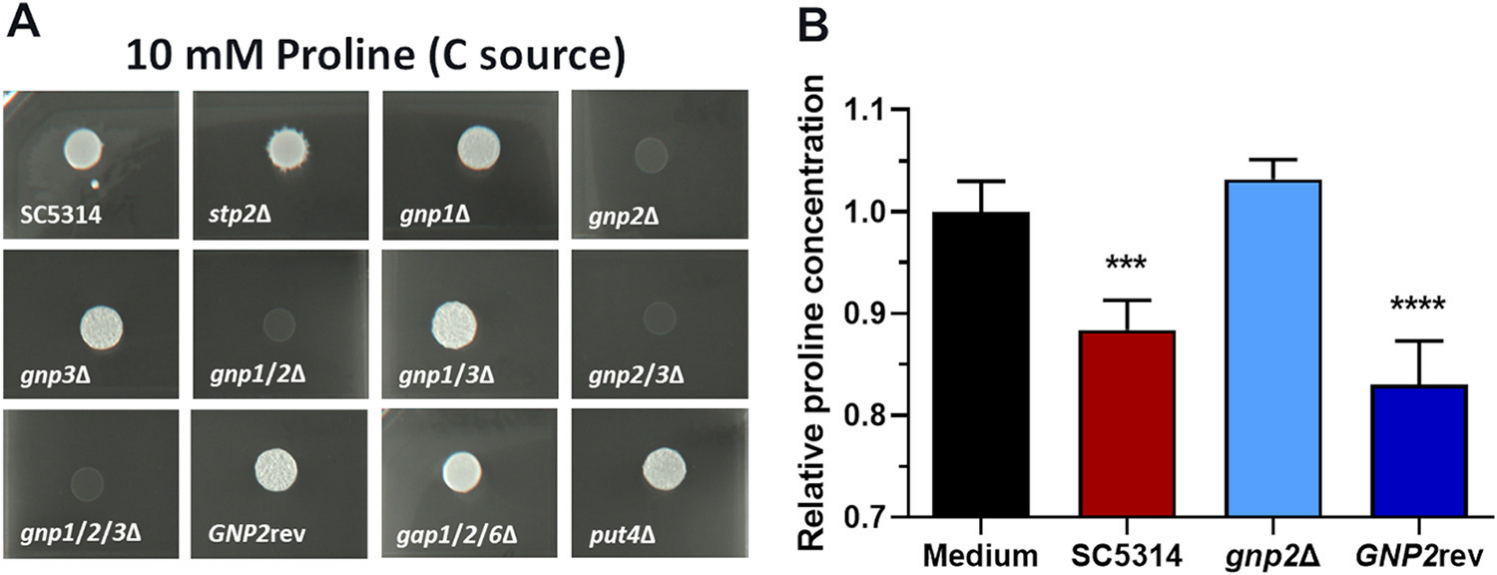
C. albicans Gnp2 is essential for proline uptake. (A) C. albicans cultures grown overnight were spotted onto agar plates containing the indicated C sources supplemented with ammonium sulfate. Plates were incubated at 37°C for 2 days (GABA) or 3 days (proline). (B) MS-based quantification of extracellular proline in spent medium containing proline as the sole carbon source (YNB, ammonium sulfate, 10 mM proline [pH 4.5]) after 6 h of incubation of the indicated strains. The proline concentration was normalized to an internal standard (10 mM 13C5 l-proline). Measurement of the medium control was performed in technical triplicates, and measurement of the spent medium was performed in five biological replicates. Significance was tested via one-way ANOVA with the medium control as the reference, indicated by asterisks (***, *P* < 0.001; ****, *P* < 0.0001).

In order to validate that exclusively Gnp2 is important for proline uptake in C. albicans, we next tested the growth of *GNP* deletion strains on proline as the sole carbon source. Additionally, we included the *put4*Δ and *gap1*Δ/*gap2*Δ/*gap6*Δ strains since ScPut4 and ScGap1 mediate proline uptake in S. cerevisiae. As expected, all strains lacking *GNP2* were strongly impaired in growth on solid (Fig. 3A) or liquid (Fig. S2C) medium with proline as the sole carbon source, whereas the other AAP deletion strains and the *GNP2* complemented strain possessed wild-type-like growth. To exclude the presence of additional proline transporters, potentially ammonium repressed, we tested growth on proline as the sole carbon and nitrogen source. Again, only the *gnp2*Δ strain displayed absent growth (Fig. S2C). Since in S. cerevisiae, Put4 is also involved in the uptake of γ-aminobutyric acid (GABA), we likewise examined all strains for growth on GABA as the sole carbon source (40). Interestingly, only strains lacking *GNP2* displayed impaired growth, more pronounced in liquid medium, whereas the *put4*Δ strain was not affected (Fig. S3).

10.1128/mBio.03142-21.3 FIG S3 C. albicans Gnp2 is required for GABA utilization. (A) C. albicans cultures grown overnight were spotted onto agar plates containing GABA as the sole carbon source and supplemented with ammonium sulfate. Plates were incubated at 37°C for 2 days. (B) The indicated C. albicans strains were grown in liquid medium containing GABA as the sole carbon source (YNB, ammonium sulfate, 10 mM GABA [pH 4.5]). Growth was assessed in a 96-well microtiter plate at 20-min intervals for 72 h. The mean of all replicates is displayed (biological duplicates and technical triplicates). Download FIG S3, TIF file, 0.9 MB. Copyright © 2022 Garbe et al. 2022 Garbe et al. https://creativecommons.org/licenses/by/4.0/ This content is distributed under the terms of the Creative Commons Attribution 4.0 International license.

Finally, to verify that Gnp2 is indeed the main proline permease, we measured proline uptake in SC5314, the *gnp2*Δ strain, and the *GNP2* complemented strain in liquid medium containing proline as the sole carbon source. After 6 h of incubation, the extracellular proline concentration was significantly reduced in the wild type and the *GNP2* complemented strain compared to the medium control but not in the *gnp2*Δ strain, proving the absence of proline uptake in this strain (Fig. 3B). Altogether, our results highlight the essential role of Gnp2 for proline uptake in C. albicans and support the notion of divergent AAP functions between this pathogenic fungus and S. cerevisiae.

### The functional difference of *GNP1* and *GNP2* is reflected in their transcriptional regulation.

Since our findings clearly show that Gnp1 and Gnp2 have distinct substrate specificities, we next examined the expression patterns of the respective genes and *GNP3* in order to detail their regulation on the transcriptional level. Given that the S. cerevisiae orthologs are regulated in either an SPS (Sc*AGP1* and Sc*GNP1*)- or an NCR (Sc*GAP1* and Sc*PUT4*)-dependent manner, we used different SPS-inducing (either glutamine or a mixture of all proteinogenic amino acids [synthetic complete [SC] medium] as the sole C source) and noninducing (either glucose [SD medium] or proline as the sole carbon source) growth conditions. To test for NCR-mediated regulation, we also measured expression in the absence of ammonium sulfate (proline or SC medium as the sole source of both nitrogen and carbon) and with proline as the sole source of nitrogen.

**FIG 4:**
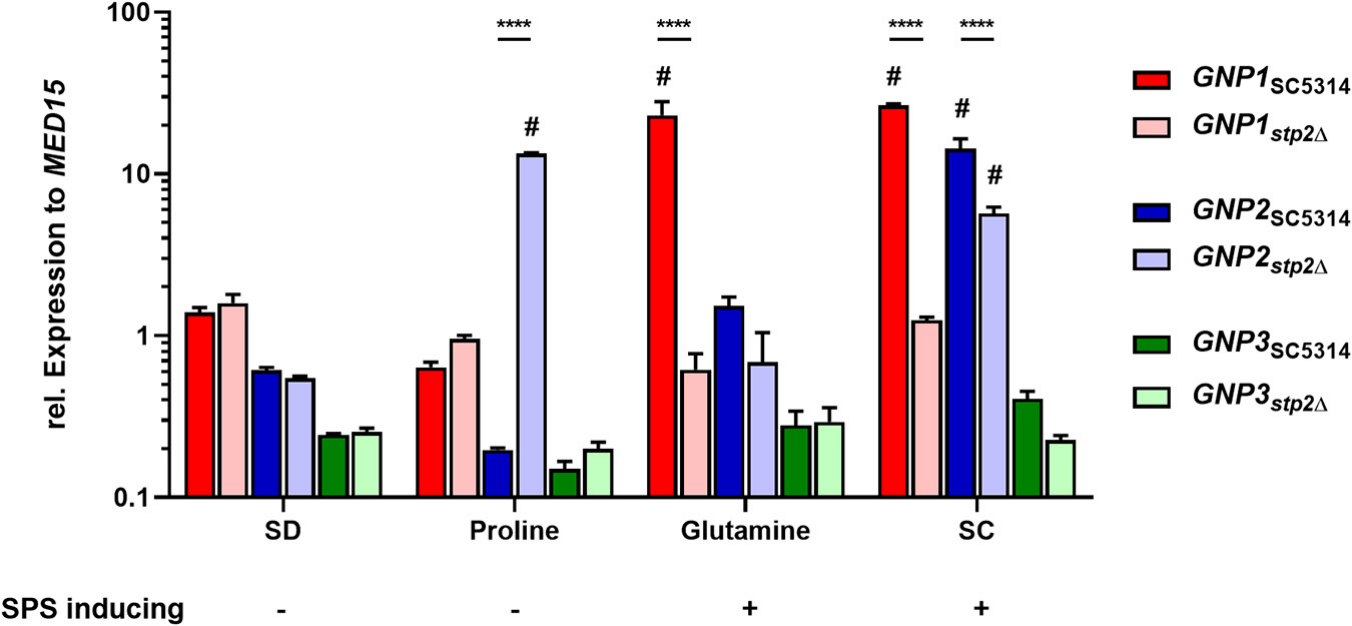
Expression of *GNP1* and *GNP2* is nutrient source and genotype dependent. YPD log-phase cells of the SC5314 wild type and the *stp2*Δ mutant were transferred to the indicated media (YNB and ammonium sulfate supplemented with either glucose, proline, or glutamine or SC medium as the sole carbon source) and incubated for 1 h at 37°C. Expression levels were measured in technical and biological triplicates, and *MED15* expression was used as the internal reference. Relative expression was calculated according to the Δ*C_T_* method. Significance was tested via two-way ANOVA between the wild type and the *stp2*Δ mutant, indicated by asterisks (****, *P* < 0.0001), and between test media and the SD medium reference for every strain, indicated by pound signs (#, *P* < 0.0001).

Under all tested conditions, the expression of *GNP3* was constant and relatively low (Fig. 4). In contrast, *GNP1* had a clear SPS-dependent expression pattern: in the wild type, the gene was induced in response to SC medium and glutamine (SPS inducing) compared to the SD control and remained unchanged in the presence of proline (SPS noninducing) or in the *stp2*Δ mutant under all conditions tested (Fig. 4). The strict SPS-dependent regulation of *GNP1* was additionally verified in a strain carrying an N-terminally truncated and, thus, constitutively active Stp2 variant (*STP2tOE*), where we noted constant induction compared to the wild type (Fig. S4A). Furthermore, *GNP1* was expressed at similar levels in SC medium without ammonium sulfate (Fig. S4B).

10.1128/mBio.03142-21.4 FIG S4 Additional expression data for *GNP1*/*2*/*3* and *PUT1*/*PUT2.* All measurements were performed in technical and biological triplicates. *MED15* expression was used as the internal reference. Relative expression was calculated according to the Δ*C_T_* method. Significance was tested via two-way ANOVA between SC5314 wild-type and mutant strains, indicated by asterisks (****, *P* < 0.0001), and between test media and the SD medium reference for every strain, indicated by pound signs (#, *P* < 0.0001). (A) Expression levels of *GNP1*/*2*/*3* were measured in the indicated media for C. albicans with either an artificially activated Put3 (*PUT3-GAD*) or Stp2 (*STP2tOE*). Media were prepared analogously to the media used for Fig. 4, and the expression levels of *GNP1*/*2*/*3* in SC5314 and the *stp2*Δ mutant were included for better comparability. (B) Expression levels of *GNP1*/*2*/*3* were measured in the indicated media for SC5314 and the *stp2*Δ mutant. Expression levels in SD medium are the same as those in Fig. 4 and are included for better comparison. Proline and SC media were prepared analogously to the media used for Fig. 4 except that ammonium sulfate was excluded. When proline was used as the sole nitrogen source, the medium was composed of YNB, 2% glucose, and 5 mM proline. (C) Expression levels of the proline catabolism genes *PUT1* and *PUT3* were measured in the SC5314, *stp2*Δ, *PUT3-GAD*, and *STP2tOE* strains. Media were prepared analogously to the media used for Fig. 4. Download FIG S4, TIF file, 0.7 MB. Copyright © 2022 Garbe et al. 2022 Garbe et al. https://creativecommons.org/licenses/by/4.0/ This content is distributed under the terms of the Creative Commons Attribution 4.0 International license.

The regulation of *GNP2* appeared more complex. First, the availability of ammonium sulfate did not influence the *GNP2* expression pattern in response to proline or SC medium, demonstrating that the expression of this gene falls outside NCR control (Fig. 4; Fig. S4B). Next, in SC5314 cells exposed to a mixture of amino acids (SC medium), *GNP2* was strongly induced compared to the amino acid-free SD medium, which was also observed in *stp2*Δ cells although to a lesser degree (Fig. 4). Therefore, we concluded SPS-independent induction of *GNP2*, which is further supported by the fact that glutamine alone failed to induce *GNP2* expression (Fig. 4). Surprisingly, the presence of proline as the sole source of carbon had no influence on *GNP2* expression levels in the wild type, while strong upregulation was visible in cells deleted of *STP2* (Fig. 4). In the presence of proline as the sole nitrogen source, we observed induction of *GNP2* in the wild type, which was further increased in the *stp2*Δ strain (Fig. S4B). Based on these observations, we hypothesized a repressing activity of Stp2 on the expression of *GNP2* under proline-rich conditions. Indeed, the expression of *GNP2* was similar to that of the wild type in the strain carrying the artificially activated Stp2 (*STP2tOE*), confirming that Stp2 negatively regulates *GNP2* expression in C. albicans in response to proline (Fig. S4A).

Recently, the transcription factor Put3 was described as a positive regulator of *GNP2* and the proline catabolism genes *PUT1* and *PUT2* (38). Indeed, we observed a significant increase of *GNP2* expression in a strain carrying an artificially activated variant of Put3 (*PUT3*-*GAD*) (Fig. S4A) (38, 41). However, no changes compared to the wild type were observed in SD medium, indicating the necessity of proline availability for this induction (Fig. S4A). Finally, we examined the expression profiles of the proline catabolism genes for potentially shared features in the regulation of proline uptake and catabolism. Interestingly, the expression patterns of both genes in response to proline resembled that of *GNP2*, displaying strong induction in the *stp2*Δ and *PUT3*-*GAD* strains (Fig. S4C). Consequently, no induction was observed for both genes in the wild-type or *STP2tOE* strain in proline compared to SD medium, confirming the repressive role for Stp2 in proline utilization (Fig. S4C).

### *GNP2* is essential for proline-induced hyphal morphogenesis in C. albicans.

A recent report showed that mitochondrial catabolism of proline acts as an inducer of C. albicans morphogenesis and is directly linked to fungal escape from macrophages (28). Thus, we examined if Gnp2-dependent uptake is also required for proline-induced filamentation *in vitro*.

**FIG 5:**
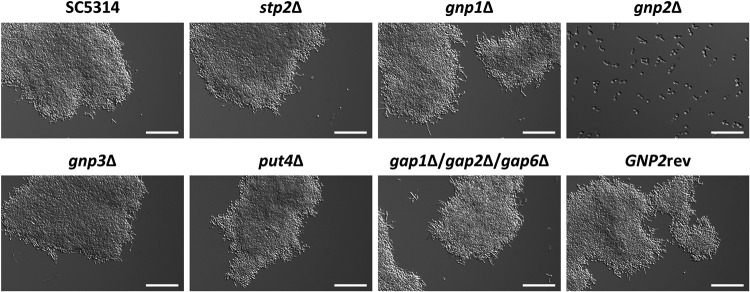
*GNP2* is required for proline-induced filamentation. C. albicans strains were tested for proline-induced filamentation in liquid medium (YNB, 0.2% glucose, 10 mM proline [pH 6]). The experiment was performed in triplicates, and representative pictures were taken after 3 h of incubation at 37°C. Bar = 100 μm (*gnp2*Δ bar = 50 μm).

Therefore, proline-induced filamentation was examined under previously reported conditions (28). As expected, the *gnp2*Δ strain remained in the yeast form, while all other tested strains displayed prominent filamentation (Fig. 5). Indeed, all strains rapidly formed dense hyphal aggregates except the *gnp2*Δ strain, which, even after prolonged incubation, displayed only sparse flocculation (Fig. S5A). Quantification of germ tube formation after a short incubation under this condition showed an absent filamentation response of the *gnp2*Δ strain to proline (Fig. S6). When proline was replaced with arginine, an amino acid that feeds into the same pathway, the *gnp2*Δ strain displayed robust filamentation indistinguishable from those of the other tested strains (Fig. S5B). Thus, we conclude that Gnp2-mediated proline uptake is required for filamentation.

10.1128/mBio.03142-21.5 FIG S5 Proline-induced flocculation and arginine-induced filamentation. (A) Macroscopic images of the indicated C. albicans strains in filamentation-inducing proline medium after 3 h and 24 h of incubation. (B) C. albicans strains were tested for arginine-induced filamentation in liquid medium (YNB, 0.2% glucose, 10 mM arginine [pH 6]). The experiment was performed in biological triplicates, and representative pictures were taken after 3 h of incubation at 37°C. Bar = 100 μm. Download FIG S5, TIF file, 1.1 MB. Copyright © 2022 Garbe et al. 2022 Garbe et al. https://creativecommons.org/licenses/by/4.0/ This content is distributed under the terms of the Creative Commons Attribution 4.0 International license.

10.1128/mBio.03142-21.6 FIG S6 Germ tube quantification after 60 min of proline-induced filamentation. C. albicans strains were tested for proline-induced filamentation in liquid medium (YNB, 0.2% glucose, 10 mM proline [pH 6]). The experiment was performed in biological triplicates. (A) Representative quantification of the germ tube and yeast proportions after 60 min of incubation from one experiment. A minimum of at least ∼200 events was counted for each strain. (B) Representative pictures after 60 min of incubation at 37°C. Germ tubes are indicated by white arrows. Bar = 50 μm. Download FIG S6, TIF file, 1.4 MB. Copyright © 2022 Garbe et al. 2022 Garbe et al. https://creativecommons.org/licenses/by/4.0/ This content is distributed under the terms of the Creative Commons Attribution 4.0 International license.

### Gnp-mediated amino acid uptake is required for cytotoxicity and fungal survival during macrophage confrontation.

**FIG 6:**
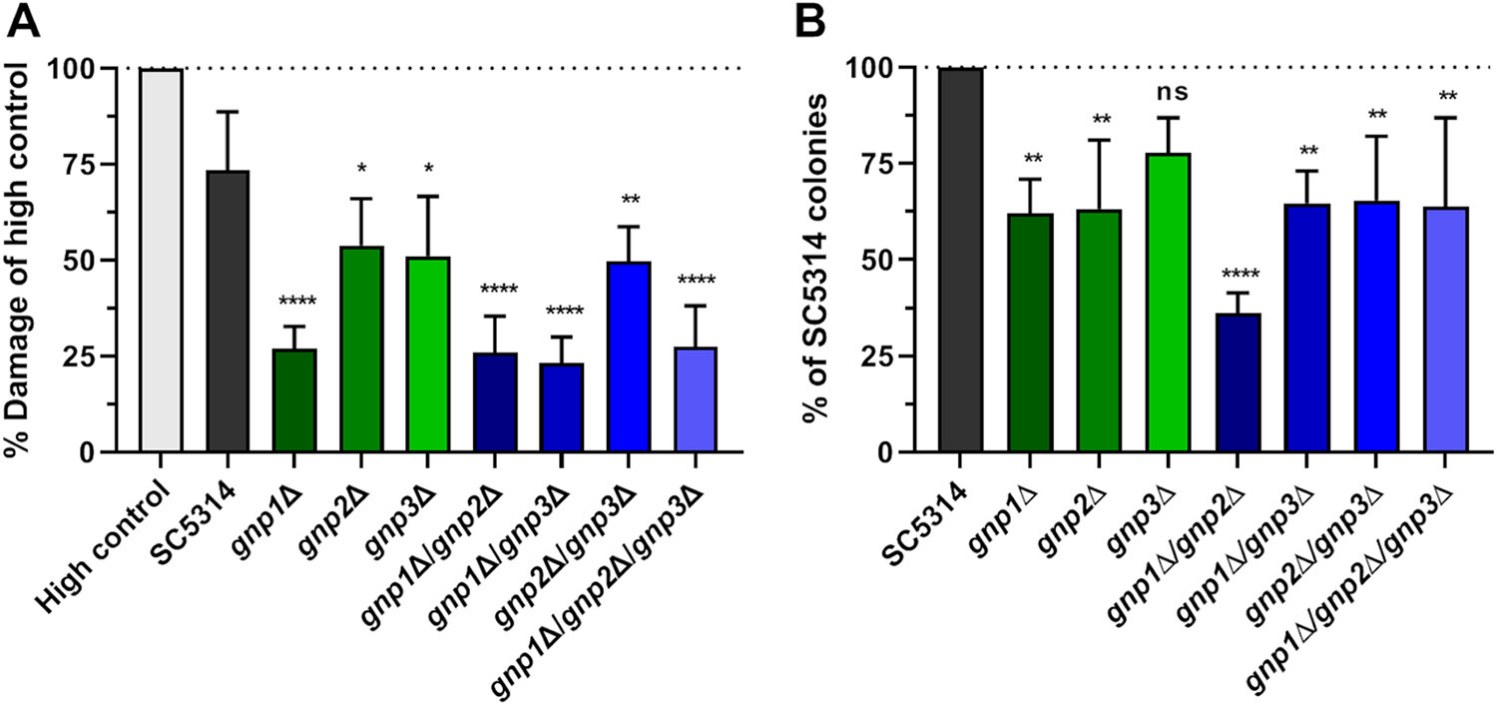
*GNP* deletion strains have impaired survival and ability to damage J774 macrophages. C. albicans cultures grown overnight were confronted with J774 macrophages for 24 h. (A) Lactate dehydrogenase (LDH) release from macrophages was measured. The damage capacity of different C. albicans strains was calculated relative to the high control (complete macrophage lysis through treatment with 5% Triton for 10 min). Significance was tested via one-way ANOVA against the wild type (*, *P* < 0.05; **, *P* < 0.01; ****, *P* < 0.0001). The experiment was performed in technical triplicates and seven biological replicates. (B) C. albicans colonies were counted after 24 h of coincubation with macrophages as the readout for fungal survival. Survival was calculated as a ratio relative to the wild type. Significance was tested via one-way ANOVA. The experiment was performed in four biological replicates.

To test the role of the Gnp1/2/3 permeases in the interaction with macrophages, we confronted strains lacking these permeases with J774 macrophages and evaluated fungal survival and the capacity to damage macrophages. Surprisingly, all three single-deletion strains were impaired in their ability to damage these immune cells, with the *gnp1*Δ strain being most affected (Fig. 6A). No additive effects were observed with strains carrying multiple deletions since the *gnp1*Δ/*gnp2*Δ, *gnp1*Δ/*gnp3*Δ, and *gnp1*Δ/*gnp2*Δ/*gnp3*Δ strains displayed damage potential similar to that of the *gnp1*Δ single mutant. Similarly, the *gnp2*Δ/*gnp3*Δ strain caused macrophage damage equally as well as the respective single-deletion strains. Of note, all tested strains displayed robust growth and filamentation in the corresponding medium (Fig. S7).

10.1128/mBio.03142-21.7 FIG S7 Growth and filamentation of *GNP* deletion strains in DMEM. (A) The growth of the indicated C. albicans strains in DMEM was assessed in a 96-well microtiter plate at 20-min intervals for 24 h. The mean of all replicates is displayed (biological duplicates and technical triplicates). (B) The filamentation of the indicated C. albicans strains was assessed in liquid DMEM. Exemplary pictures after 4 h of incubation are shown. Bar = 100 μm. Download FIG S7, TIF file, 1.2 MB. Copyright © 2022 Garbe et al. 2022 Garbe et al. https://creativecommons.org/licenses/by/4.0/ This content is distributed under the terms of the Creative Commons Attribution 4.0 International license.

The reduced ability of the permease mutants to damage J774 macrophages was also reflected in the impaired fungal survival following confrontation with these immune cells. However, some notable differences were observed (Fig. 6B). For both the *gnp1*Δ and *gnp2*Δ strains, survival was about 60% of that of the wild-type control. In the *gnp3*Δ strain, survival was also reduced albeit not significantly. However, the *gnp1*Δ/*gnp2*Δ strain showed an even further decrease in survival after macrophage confrontation (∼35%), whereas the *gnp1*Δ/*gnp3*Δ and *gnp2*Δ/*gnp3*Δ strains had survival rates comparable to those of strains with deletions of either *GNP1* or *GNP2*, respectively. Oddly, the triple-deletion strain displayed higher survival rates than the *gnp1*Δ/*gnp2*Δ strain (Fig. 6B). Altogether, these results demonstrate an important role of Gnp1- and Gnp2-mediated amino acid uptake for C. albicans-macrophage interactions.

### Gnp2-mediated proline uptake is beneficial for C. albicans ROS resistance.

The reduced capacity to damage macrophages and to survive the immune cell confrontation of *GNP* deletion strains was not linked to filamentation defects under these conditions since all strains were equally capable of hypha formation upon phagocytosis (data not shown). Macrophages use a variety of mechanisms to ensure restricted growth and the eradication of the engulfed microbes, including the oxidative burst (42). Thus, we examined if Gnp1/2/3 are required for fungal adaptation to reactive oxygen species (ROS).

**FIG 7:**
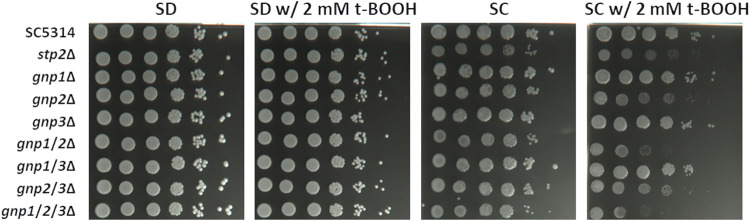
Gnp2 and, to a lesser degree, Gnp1 are important for fungal resistance against oxidative stress. Serial dilutions (OD of 1, 10^−1^, 10^−2^, 10^−3^, 10^−4^, and 10^−6^) of C. albicans cultures grown overnight were spotted onto plates with the indicated medium in either the presence or absence of the ROS stress inducer t-BOOH. The plates were incubated for 2 days at 37°C. All assays were performed in biological triplicates. Representative pictures are shown.

The ROS inducer *tert*-butyl hydroperoxide (t-BOOH) was added to two different media: SC medium containing all proteinogenic amino acids and SD minimal medium without any amino acids as the control. As expected, when no amino acids were present, none of the AAP deletion strains exhibited increased sensitivity to ROS stress compared to the wild type (Fig. 7). However, in SC medium, the *gnp2*Δ strain displayed markedly increased sensitivity to t-BOOH, a phenotype similar to that of the *stp2*Δ strain, indicating a beneficial effect of amino acid uptake for the fungus when exposed to ROS stress. Interestingly, the *gnp1*Δ/*gnp2*Δ and *gnp1*Δ/*gnp2*Δ/*gnp3*Δ strains showed further increases in sensitivity, whereas the *gnp1*Δ and *gnp3*Δ strains remained unaffected. Thus, amino acid transport via Gnp2 and, to a lesser degree, Gnp1 appears important for the adaptation of C. albicans to oxidative stress.

### Gnp-mediated amino acid uptake is not critical for full virulence in a murine model of disseminated candidiasis.

**FIG 8:**
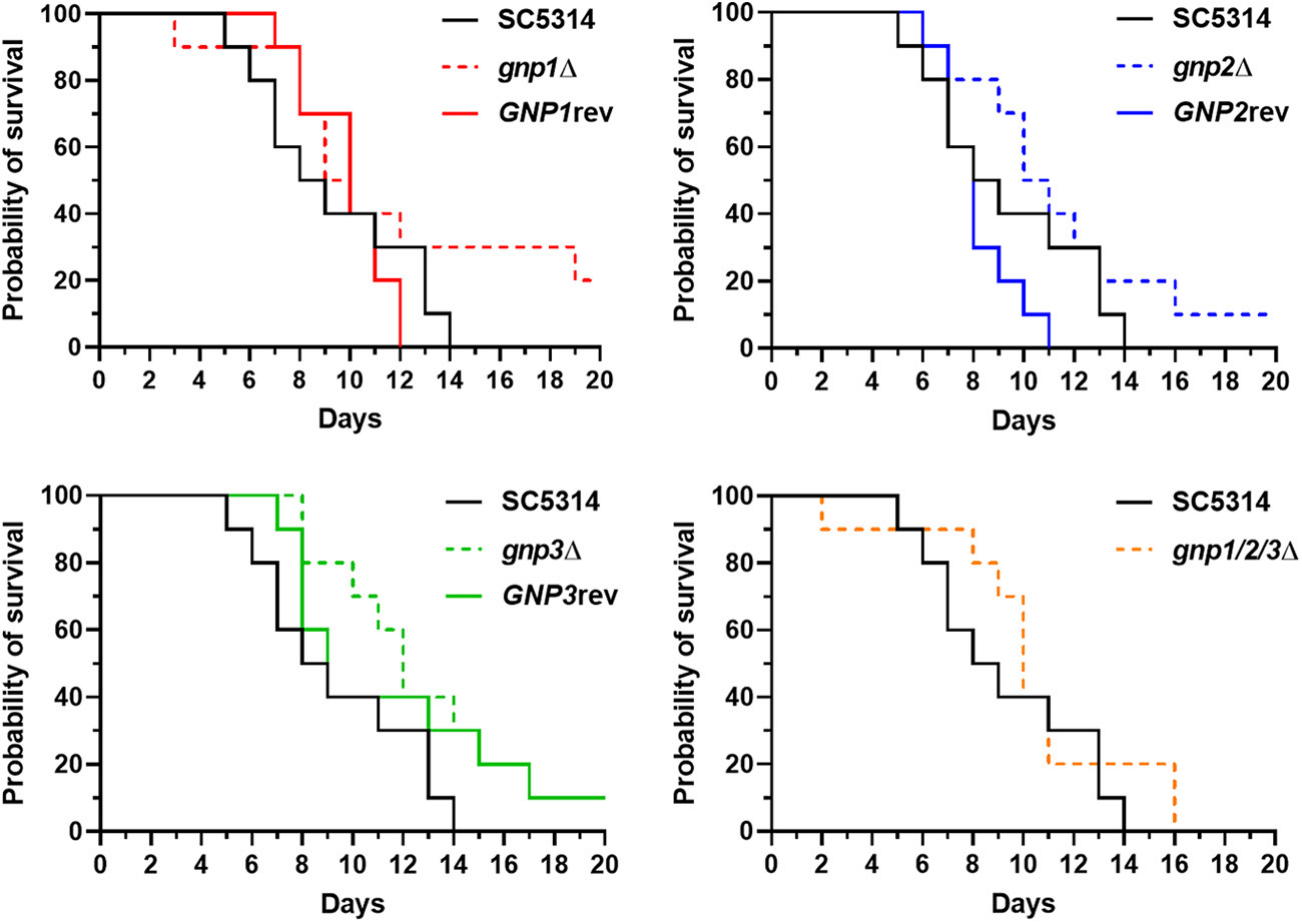
Survival rates of mice after C. albicans tail vein injection. C. albicans cells of the indicated genotype were injected into the lateral tail vein of mice and monitored for survival. The data presented represent the results of one experiment with a single wild-type control but are split into four panels for ease of visualization. Significance was tested via a log rank (Mantel-Cox) test.

To assess whether Gnp1/2/3-mediated amino acid uptake is required for full fungal virulence *in vivo*, we tested the *gnp1*Δ, *gnp2*Δ, and *gnp3*Δ strains; the corresponding revertant strains; and the *gnp1*Δ/*gnp2*Δ/*gnp3*Δ strain in a murine model of disseminated candidiasis. Surprisingly, despite the prominent *in vitro* phenotypes, no significant reduction in virulence was seen (Fig. 8). However, a slight trend toward increased survival was visible for mice infected with the *gnp1*Δ, *gnp2*Δ, and *gnp3*Δ single mutants.

## DISCUSSION

Metabolic flexibility and the ability to quickly react to environmental changes are two closely intertwined characteristics of paramount importance for C. albicans and its successful lifestyle as an opportunistic pathogen (8, 9). A key role in this context is the utilization of amino acids, which affects fungal virulence in various ways (21). In this work, we focused on AAP-mediated uptake of amino acids and especially proline, a particularly good source of energy, as the oxidative phosphorylation of 1 mol proline yields approximately 30 mol ATP, nearly equivalent to glucose (43). While proline promotes C. albicans growth not only as the sole nitrogen source but also as a source of carbon, in S. cerevisiae proline utilization is limited to nitrogen utilization only (38, 44). This substantial difference suggests that the mechanisms of uptake and cellular fate of this amino acid differ between the two microorganisms. While the AAPs involved in S. cerevisiae proline uptake are well defined, the sensing and transport of extracellular proline in C. albicans have not been studied.

Here, we demonstrate that C. albicans possesses 10 orthologs of the four proline-transporting AAPs in S. cerevisiae (ScAgp1, ScGnp1, ScGap1, and ScPut4). These orthologs are involved in the uptake of various amino acids, but only Gnp2 mediates proline uptake in C. albicans. Indeed, the C. albicans genome encodes six ScGap1 orthologs, but the deletion of none of them affected the sensitivity to the proline analog (30). The strain with the triple deletion of *GAP1*, *GAP2*, and *GAP6* was also not impaired in growth on proline, although Gap2 and Gap6 were previously reported to complement proline utilization in an uptake-deficient S. cerevisiae strain (30). Furthermore, Gnp1 appears to play an important role in the uptake of asparagine, glutamine, tryptophan, and, to a lesser extent, threonine and methionine. The physiological relevance of Put4 remains unclear since a C. albicans strain lacking *PUT4* was not impaired in proline but likely involved in lysine uptake. Moreover, Put4 appeared dispensable for the uptake of GABA, unlike ScPut4 (40). Instead, this function is mediated by Gnp2, which is required for growth on GABA as the sole carbon source, while it was expendable during growth on GABA as the sole nitrogen source (data not shown). Taken together, our results show that Gnp1 and Gnp2 are functionally distinct in C. albicans: Gnp2 is a specialized proline and GABA transporter, whereas Gnp1 has broader substrate specificity, comparable to those of ScAgp1 and ScGnp1 (45–49).

This specialization is further exemplified by the diverged transcriptional regulation of *GNP1* and *GNP2*. While Sc*AGP1* and Sc*GNP1* are both regulated in an SPS-dependent manner, in C. albicans, only *GNP1* is positively regulated by Stp2 (37). This correlates well with the observed substrate specificity for Gnp1, which, aside from tryptophan, coordinates the uptake of only SPS-stimulating amino acids (28). In contrast, the regulation of *GNP2* is more complex. Since it was strongly induced in response to a mixture of amino acids in the wild-type and *stp2*Δ strains, although slightly lower, we concluded mainly SPS-independent induction. However, *GNP2* was strongly induced in *stp2*Δ cells in the presence of proline as the sole carbon source. This observation was quite unexpected given that under this condition, the transcription factor Stp2 is considered to be retained in the cytosol (28, 33). Furthermore, *GNP2* was not induced in a strain with a constitutively active variant of Stp2. Interestingly, the same observations were made for the proline catabolism genes *PUT1* and *PUT2*, which shared the *GNP2* expression pattern under all tested conditions, suggesting that proline uptake and catabolism are similarly regulated. These results strongly point toward Stp2-mediated repression of proline utilization, the underlying mechanisms of which remain unclear. One possible connection is Dal81, a transcription factor involved in GABA utilization in C. albicans, shown to interact with full-length and processed Stp2 in S. cerevisiae to enhance its transcriptional activity (50, 51). Another factor is Put3, previously described as a positive regulator of proline utilization in C. albicans (38). Indeed, we observed that a strain carrying an artificially activated variant of Put3 had significantly elevated transcript levels of *PUT1*, *PUT2*, and *GNP2* in the presence of proline. While the dependence of proline availability for Put3 activity is a well-established mechanism for S. cerevisiae, the potential cross talk of Stp2- and Put3-mediated gene regulation in C. albicans requires further investigation (52).

The outstanding nature of proline uptake in C. albicans inevitably prompts the question of why such specialization has evolved. Since proline is a valuable source of carbon and nitrogen for this pathogenic fungus, a simple explanation would be to allow rapid adaptation to the proline-rich niches of the human host. This amino acid is especially enriched in the extracellular matrix in the structural protein collagen, composed of up to 25% proline/hydroxyproline (53). There, it might serve as an easily accessible energy source during invasive growth. Additionally, proline catabolism is directly linked to pathogenicity, as it triggers hyphal growth and is required for the capacity to damage macrophages (28). Accordingly, we found that proline-induced filamentation *in vitro* is dependent on Gnp2. However, *gnp2*Δ cells were fully capable of forming filaments within macrophage phagosomes. It is feasible that the uptake of arginine or ornithine compensates for the impaired ability of the *gnp2*Δ strain to import proline during phagocytosis, resulting in the activation of the same filamentation pathway (18, 27, 28).

Despite the retained ability to filament within the macrophage phagosome, the strains deleted of *GNP2* were impaired in damaging these immune cells and had impaired survival following macrophage confrontation. Interestingly, previous studies also reported that *GNP2* is induced following confrontation with neutrophils or macrophages (22, 23). Since these immune cells confront C. albicans with a combination of factors, this could indicate the physiological relevance of proline uptake in adaptation to host-imposed stresses. This notion is supported by the significantly increased sensitivity to oxidative stress of strains lacking *GNP2*. Indeed, proline plays a prominent role in the stress adaptation of many organisms ranging from humans to plants and microbes (43). Due to its special structure, this amino acid fulfills a variety of functions: it acts not only as a direct ROS scavenger, detoxifying hydroxyl radicals or singlet oxygen, but also as a chemical chaperone stabilizing redox enzymes. It further contributes to the cellular glutathione pool, which is essential in detoxifying ROS (43). While the role of proline-mediated stress adaptation has not been investigated in C. albicans, functional proline catabolism was previously described as being essential for ROS homeostasis and virulence in Cryptococcus neoformans (54). Furthermore, the proline-mediated stress response is thoroughly studied in the model yeast S. cerevisiae (55, 56). Although the underlying mechanisms are not fully understood so far, proline homeostasis and forced accumulation are beneficial in resistance to environmental stresses like oxidation, desiccation, and freezing, partially due to the positive influence on membrane and protein stability.

In this context, the importance of Gnp2 in GABA utilization is especially interesting. The GABA shunt is a highly conserved pathway in most organisms and closely intertwined with proline metabolism and the TCA cycle. In S. cerevisiae this pathway was shown to be involved in thermotolerance by reducing ROS production and in the regulation of the replicative life span (57, 58). GABA accumulation also accompanies and influences stress responses in several plants (59). The physiological importance of GABA in C. albicans remains poorly investigated, with reports linking Gpt1, a putative GABA/polyamine transporter, and the transcription factor Uga3 to the utilization of this host-relevant nutrient (51, 60). However, as dual-specificity GABA/proline transporters have been previously reported for other microorganisms, for instance, Bacillus subtilis, it is feasible that similar transport systems could exist in other pathogenic organisms like C. albicans (61).

Despite the impairments in growth, filamentation, and macrophage interactions, we observed no significant reduction in the virulence of *GNP1*/*2*/*3* deletion strains in a murine model of disseminated candidiasis. This suggests that either certain compensatory mechanisms or residual unspecific proline uptake is sufficient to support the growth and pathogenicity of the strains in this infection model. Another possibility is that proline uptake is not critical for systemic infection but rather more relevant during the commensal growth of C. albicans. In its commensal niches, like the gastrointestinal tract, the fungus is subjected to mutualistic and competitive interactions with the associated microbiota. Recently, the metabolic interactions of C. albicans and gut bacteria were modeled using genome-scale metabolic models, revealing that proline is one of the few metabolites readily consumed by bacteria, which negatively influences fungal growth (62). Since *GNP2* was found to be induced during C. albicans colonization of the gastrointestinal tract, the presence of a high-specificity proline permease might provide a competitive edge in this environment (63).

In conclusion, our results clearly demonstrate the presence of specialized proline uptake in C. albicans and its relevance for fungal physiology. The precise reason for this specialization, its advantages over redundant uptake, and the underlying mechanisms for sensing and regulation require further investigations. These results, together with the role of GABA-mediated cellular processes, should lead to a more comprehensive understanding of C. albicans pathogenesis.

## MATERIALS AND METHODS

### C. albicans cultivation.

All C. albicans strains used in this study are listed in Table S3A in the supplemental material. Unless indicated otherwise, C. albicans was routinely cultured in yeast extract-peptone-dextrose (YPD) medium (1% yeast extract, 2% peptone, and 2% glucose, solidified with 2% agar for plates) at 30°C. Precultures were grown overnight with shaking at 180 rpm and washed twice with sterile distilled water (dH_2_O) prior to subsequent experiments.

10.1128/mBio.03142-21.10 TABLE S3 Strains, oligonucleotides, and UHPLC gradient used in this study. Download Table S3, XLSX file, 0.01 MB. Copyright © 2022 Garbe et al. 2022 Garbe et al. https://creativecommons.org/licenses/by/4.0/ This content is distributed under the terms of the Creative Commons Attribution 4.0 International license.

### Strain construction.

The C. albicans wild-type strain SC5314 was used for all gene deletions. For the generation of AAP mutants, a modified CRISPR-Cas9 approach was used to simultaneously delete both alleles of the gene of interest (64). Briefly, Cas9 was PCR amplified from pV1093. Specific guide RNAs were chosen and PCR amplified to be expressed under the control of the SNR52 promoter. Repair constructs containing the *SAT1-FLP1* cassette were PCR amplified from pSFS2 (65), using long primers with 50-bp homologous flanking fragments specific to the target gene. All PCR products were column purified, and 500 ng of each fragment (Cas9, the gene-specific single guide [sgRNA], and the gene-specific repair construct) was used to electroporate C. albicans (66). Transformants were selected on YPD-nourceothricin (YPD-Nat) medium, and the correct gene deletion was confirmed by PCR. The correct clones were rendered Nat sensitive by growing them overnight on yeast extract-peptone-maltose (YPM) (2% maltose) to excise the *SAT1-FLP1* cassette. For complementation, the gene of interest was amplified from genomic DNA and fused by overlap extension PCR to the *SAT1* product from pSFS2, including 50 bp of homology upstream and downstream of the target gene. A guide RNA targeting the FLP recombination target (FRT) scar left behind after *SAT1-FLP1* excision was PCR amplified. The mutant strain to be complemented was electroporated with 500 ng of each PCR fragment (Cas9, FRT sgRNA, and the target gene fused to *SAT1*). Transformants were selected on YPD-Nat, and the correct gene complementation was confirmed by PCR. The generation of a constitutively active Stp2, through truncation of amino acid residues 2 to 100, was performed analogously to the previously described generation of the *STP2* overexpression strain (67). The truncated variant of *STP2* was synthesized by Genewiz.

### Growth assays. (i) Solid medium.

C. albicans cultures grown overnight were set to an optical density at 600 nm (OD_600_) of 0.5 in sterile dH_2_O, and 5 μL was spotted per strain. All plates contained 0.17% yeast nitrogen base (YNB) without ammonium sulfate and amino acids and 2% purified Bacto agar (BD, NJ, USA). For growth on defined carbon sources (10 mM Pro or GABA), the plates were supplemented with 0.5% ammonium sulfate. The plates were incubated at 37°C for 2 to 3 days, and images were taken with a ProtoCol2 colony counter (Synbiosis, UK).

### (ii) Liquid medium.

Growth assays in liquid medium were performed in 96-well microplates using an Infinite M Nano microplate reader (Tecan, Switzerland). If not indicated otherwise, C. albicans cultures grown overnight were set to an OD_600_ of 2 in sterile dH_2_O, and 2 μL was added to 198 μL test medium per well for a final OD of 0.02. The OD_600_ was subsequently measured every 20 min for 24 h or 72 h at 37°C with 10 s of shaking prior to each measurement. All measurements were performed in technical triplicates and biological duplicates. Growth was measured as the area under the curve (AUC) (GraphPad Prism 8.4.3, trapezoidal method; baseline at 0.02).

To assess growth on single amino acids as the sole nitrogen source, base medium composed of 0.17% YNB and 2% glucose was supplemented with 5 mM the indicated nitrogen source, except for tyrosine and ammonium sulfate, with 1.5 mM and 0.5%, respectively. The initial pH was adjusted to 5. The growth of SC5314 on single amino acids was calculated as a ratio of the growth on ammonium sulfate. The growth of AAP deletion strains was calculated as the ratio of SC5314 growth under the respective conditions.

Resistance to toxic amino acid analogs (Sigma-Aldrich, MO, USA) (Table S2A) was monitored through their addition to SD medium (0.17% YNB, 0.5% ammonium sulfate, 2% glucose [pH 5]). Growth in the presence of toxic analogs was calculated relative to growth in SD medium alone. Due to the various degrees of SC5314 growth inhibition through the individual analogs, the growth of the particular AAP deletion strains was calculated as a percentage of wild-type inhibition where the effect for each analog on the wild type is set to 100%. As a consequence, 100% wild-type inhibition for an AAP deletion strain indicates growth inhibition similar to that of SC5314, while 0% wild-type inhibition indicates the unaffected growth of an AAP deletion strain in the presence of an analog. The following formula was used for calculation:

$$\%\text{wild-type inhibition }=\frac{\left[100-\left(\frac{{\text{AUC mutant}}_{\text{analog}}\times 100}{{\text{AUC mutant}}_{\text{SD}}}\right)\right]\times \text{100}}{100-\left(\frac{{\text{AUC SC5314}}_{\text{analog}}\times 100}{{\text{AUC SC5314}}_{\text{SD}}}\right)}$$

### Quantification of proline uptake.

C. albicans proline uptake was quantified as follows. C. albicans cultures grown overnight were used to inoculate 10 mL test medium (0.17% YNB, 0.5% ammonium sulfate, 10 mM proline [pH 4.5]), which was subsequently incubated at 37°C for 6 h. One milliliter of culture per sample was sampled, and after centrifugation, 100 μL of the supernatant was supplemented with 13C5 l-proline (Eurisotop, MA, USA) for a final concentration of 10 mM as the internal reference.

Ultrahigh-performance liquid chromatography (UHPLC) coupled with high-resolution mass spectrometry (MS) was carried out using a Thermo (Bremen, Germany) UltiMate HPG-3400 RS binary pump and a WPS-3000 autosampler, which was set to 10°C and was equipped with a 25-μL injection syringe and a 100-μL sample loop. The column was kept at 25°C within the column compartment of the TCC-3200 system. A Thermo Accucore C_18_ reverse-phase (RP) chromatography column (100 by 2.1 mm, 2.6 μm) was used, using the gradient listed in Table S3C, at a constant flow rate of 0.4 mL/min. Eluent A was water with 2% acetonitrile and 0.1% formic acid. Eluent B was pure acetonitrile.

Mass spectra were recorded with a Thermo QExactive plus orbitrap mass spectrometer coupled to a heated electrospray ionization (HESI) source. The column flow was switched at 0.5 min from waste to the mass spectrum and at 11.5 min again back to the waste, to prevent source contamination. For monitoring, two full-scan modes were selected with the following parameters: positive polarity, a scan range of *m/z* 80 to 1,200, a resolution of 70,000, an automatic gain control (AGC) target of 3 × 10^6^, and a maximum ion time of 200 ms. General settings were as follows: a sheath gas flow rate of 60, an auxiliary gas flow rate of 20, a sweep gas flow rate of 5, a spray voltage of 3.0 kV, a capillary temperature of 360°C, an S-lens radio frequency level of 50, an auxiliary gas heater temperature of 400°C, and an acquisition time frame of 0.0 to 11.5 min. For negative mode, all values were kept instead of the spray voltage, which was set to 3.3 kV.

Peak detection and integration were carried out using Thermo Scientific Xcalibur 3.0.63 Quan Browser software with the following settings: a mass tolerance of 5 ppm, a mass precision of 4 decimals, and the compounds proline (calculated for C_5_H_10_NO_2_ as [M + H]^+^ of 116.0706) and labeled proline (calculated for ^13^C_5_H_10_NO_2_ as [M + H]^+^ of 121.0874). A retention time of 0.66 min was set, including the window, which was set to 30 s; the signal was the extracted ion chromatogram (XIC) from the positive full-scan experiment; the peak detection algorithm was ICIS (smoothing = 1; baseline window = 40; area noise factor = 5; peak noise factor = 10); and the peak detection method was the highest peak, with a minimum peak height (signal-to-noise [S/N] ratio) of 3. Area ratios of each analyte relative to the internal standard (IS) were determined, and the analyte concentration of each sample was calculated relative to the average of the medium control. For statistical analysis, Microsoft Excel 365, version 2108, was used. Error bars were calculated using the confidence interval with 95% probability. Significance was tested via one-way analysis of variance (ANOVA) using GraphPad Prism 8.4.3 with the medium control as the reference.

### ROS stress assays.

To determine ROS sensitivity *in vitro*, SD and SC (0.17% YNB, 0.5% ammonium sulfate, 1% complete SC medium without ammonium sulfate [Biomol, Germany]) media were solidified with 2% purified Bacto agar (BD) and the indicated concentrations of *tert*-butyl hydroperoxide (Luperox; Sigma-Aldrich) added. C. albicans cultures grown overnight were set to an OD_600_ of 1 in sterile dH_2_O, 10-fold serial dilutions were prepared (OD of 1, 10^−1^, 10^−2^, 10^−3^, 10^−4^, and 10^−5^), and 5 μL from each dilution was spotted. The plates were subsequently incubated for 2 days at 37°C, and images were taken with a ProtoCol2 colony counter (Synbiosis, UK). The experiments were performed in biological triplicates.

### Filamentation experiments.

Proline- and arginine- or Dulbecco’s modified Eagle’s medium (DMEM)-induced filamentation in liquid medium was analyzed as follows: C. albicans cultures grown overnight were inoculated into 30 mL of test medium (0.17% YNB, 0.2% glucose, and 10 mM proline or arginine [pH 6] or DMEM [catalog number 41965; Thermo Fisher Scientific]) at a final OD of 0.3 and incubated at 37°C with shaking at 180 rpm (28). Samples were fixed at the indicated time points with 4% Histofix (Carl Roth, Germany), and morphology was assessed via differential interference contrast (DIC) imaging with a Zeiss AxioObserver Z.1 microscope. The experiments were performed in biological triplicates, and exemplary pictures are shown. For the quantification of proline-induced germ tube formation after 60 min, a minimum of 185 cells per strain from one representative experiment were counted and classified as either germ tube or yeast, and the relative proportions of both groups were calculated.

### RNA extraction and qRT-PCR.

Gene expression levels were measured via quantitative reverse transcription PCR (qRT-PCR) under the following growth conditions: SD medium with proline or glutamine as the sole carbon source (0.17% YNB, 0.5% ammonium sulfate, 10 mM proline or glutamine [pH 5]), proline as the sole carbon source without ammonium sulfate, or proline as the sole nitrogen source (0.17% YNB, 5 mM proline, 2% glucose [pH 5]) and SC medium with or without ammonium sulfate (0.17% YNB with or without 0.5% ammonium sulfate and 1% complete SC medium without ammonium sulfate [Biomol, Germany] [pH 5]). C. albicans cultures grown overnight were diluted to an OD of 0.3 in 50 mL YPD and incubated at 37°C with shaking at 180 rpm until early log phase was reached (OD_600_ = 1). Cells were collected, washed twice with dH_2_O, and used to inoculate 10 mL test medium at an OD of 0.3. The cultures were incubated at 37°C for 60 min, and cells were harvested, snap-frozen in liquid nitrogen, and stored at −80°C until further processing. For total RNA isolation, cells were resuspended in 440 μL acetate EDTA buffer (50 mM sodium acetate, 10 mM EDTA) with 10% SDS, and 440 μL of acid-phenol-chloroform with isoamyl alcohol (Ambion, Germany) was added. The suspension was thoroughly mixed, incubated for 5 min at 65°C, frozen at −20°C for 30 min, and subsequently thawed at 65°C for 5 min. The phases were separated via 4 min of centrifugation at 16,000 × *g*, the upper aqueous phase was transferred, and a 1/10 volume of sodium acetate (pH 5.3) and 1 volume of 2-propanol were added to precipitate the RNA for at least 30 min at −20°C. The supernatant was discarded after 10 min of centrifugation at 12,000 × *g*, and the RNA pellet was washed twice with 80% ethanol and finally solved in 300 μL nuclease-free H_2_O. The quality and quantity of the RNA were assessed using 2100 bioanalyzer (Agilent Technologies, CA, USA) and Nanodrop (Thermo Fisher Scientific, MA, USA) measurements. qRT-PCR was performed using Brilliant III Ultra-Fast SYBR green qRT-PCR master mix (Agilent Technologies) on a Stratagene Mx3005P system (Agilent Technologies) with 100 ng/μL RNA as the template. The expression levels were calculated according to the Δ*C_T_* method using *MED15*, a subunit from the RNA polymerase II mediator complex, as the internal reference (68). The oligonucleotides used are listed in Table S3B.

### Macrophage infection assays.

Measurements of fungal macrophage cytotoxicity and survival were performed as previously described (69). Solely, the survival rates of AAP deletion strains were calculated relative to the wild type as follows:

$$\%\text{ survival }=\frac{{\text{colonies}}_{\text{mutant}}}{{\text{colonies}}_{\text{SC5314}}}\times 100$$

### Mouse model of disseminated candidiasis.

C. albicans strains were grown in YPD to log phase, washed, and resuspended in phosphate-buffered saline (PBS). A total of 2 × 10^5^ cells were injected into the lateral tail vein of a total of 10 female 7- to 9-week-old ICR mice per strain. All mice were monitored 3 times per day and euthanized when moribund. All experiments were approved by the Animal Welfare Committee of the University of Texas Health Science Center at Houston. Survival curves were tested for significant differences via a log rank (Mantel-Cox) test.
