# Supplementary figures and images for: GNP2 Encodes a High-Specificity Proline Permease in Candida albicans
Source: mBio. 2022 Jan 25;13(1):e03142-21. doi: 10.1128/mbio.03142-21 (PMC8787483; doi:10.1128/mbio.03142-21)

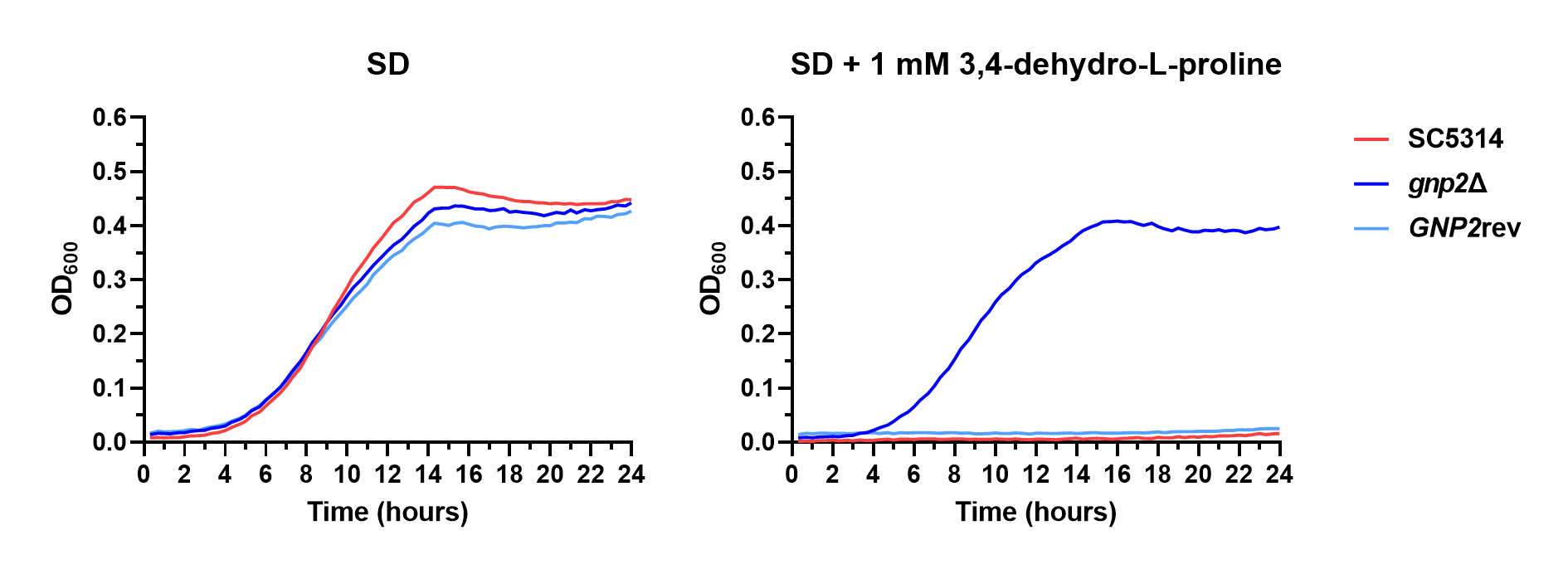

Supplement: FIG S1 [file mbio.03142-21-sf001.tif]

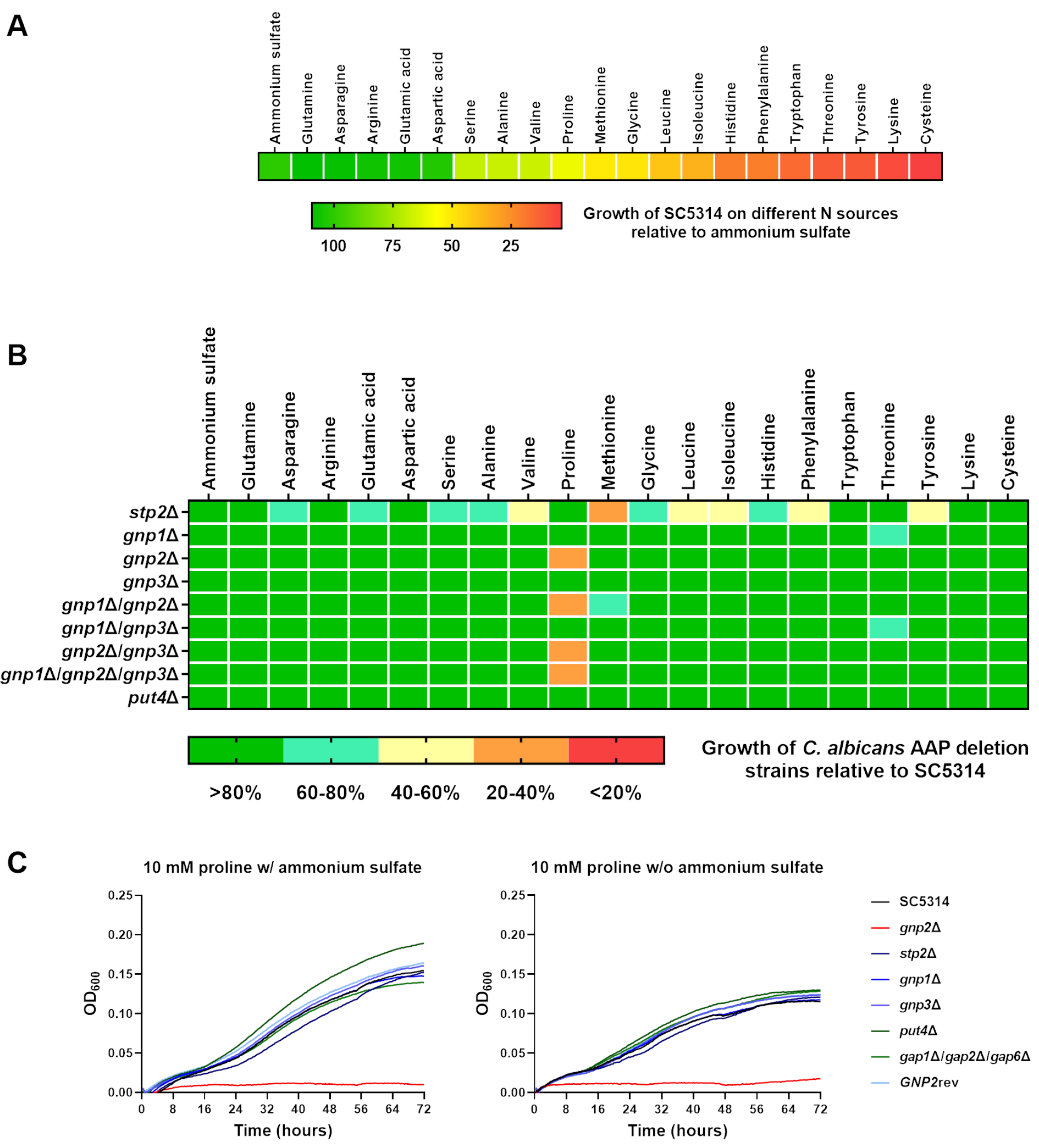

Supplement: FIG S2 [file mbio.03142-21-sf002.tif]

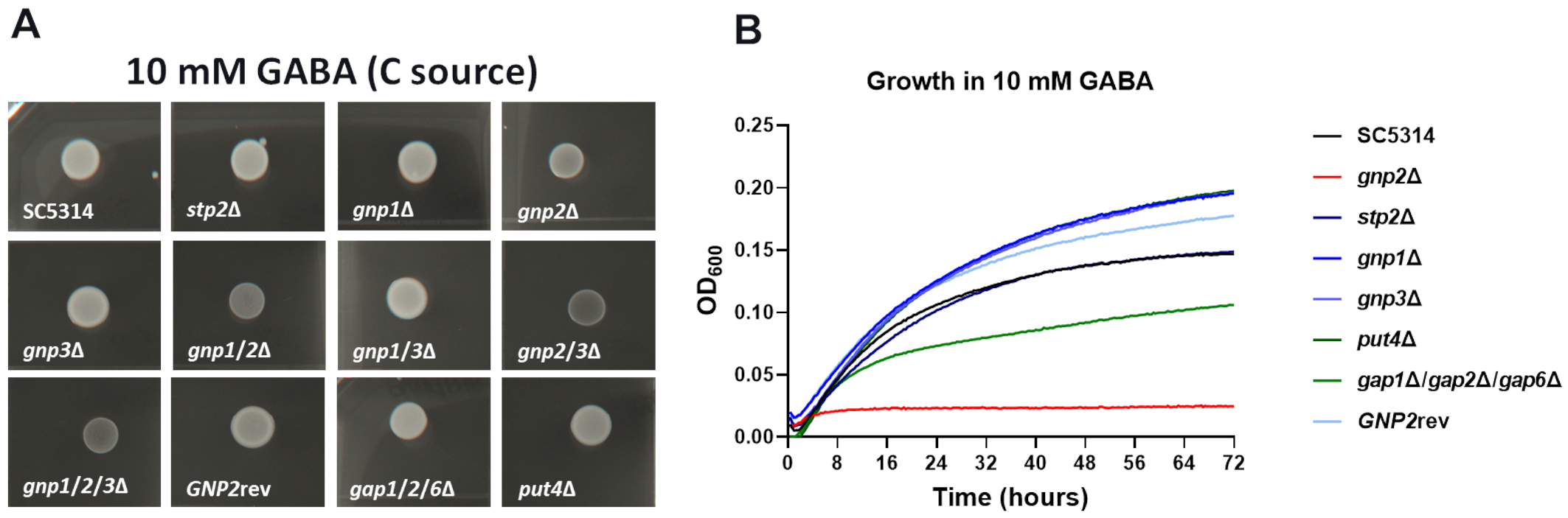

Supplement: FIG S3 [file mbio.03142-21-sf003.tif]

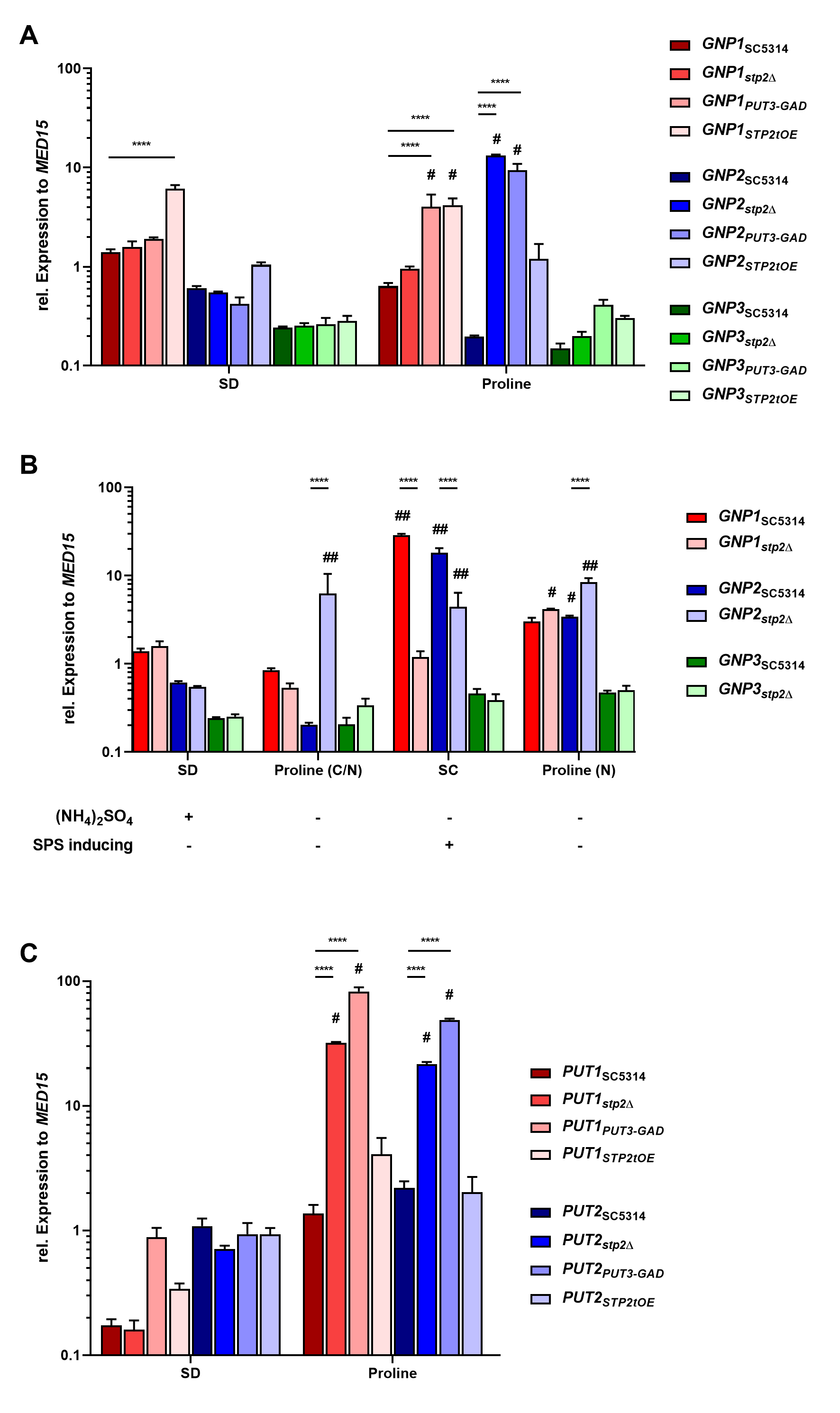

Supplement: FIG S4 [file mbio.03142-21-sf004.tif]

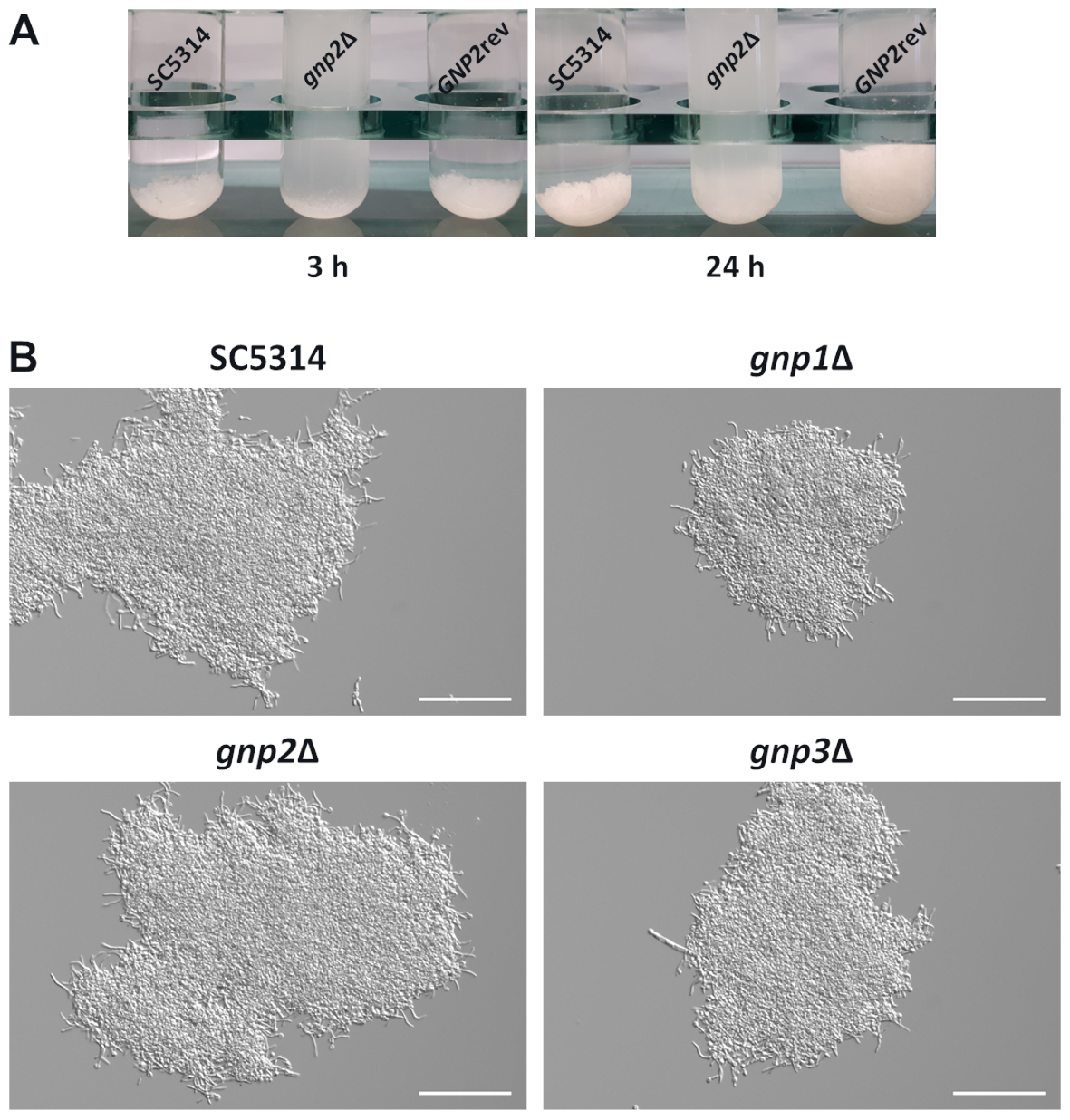

Supplement: FIG S5 [file mbio.03142-21-sf005.tif]

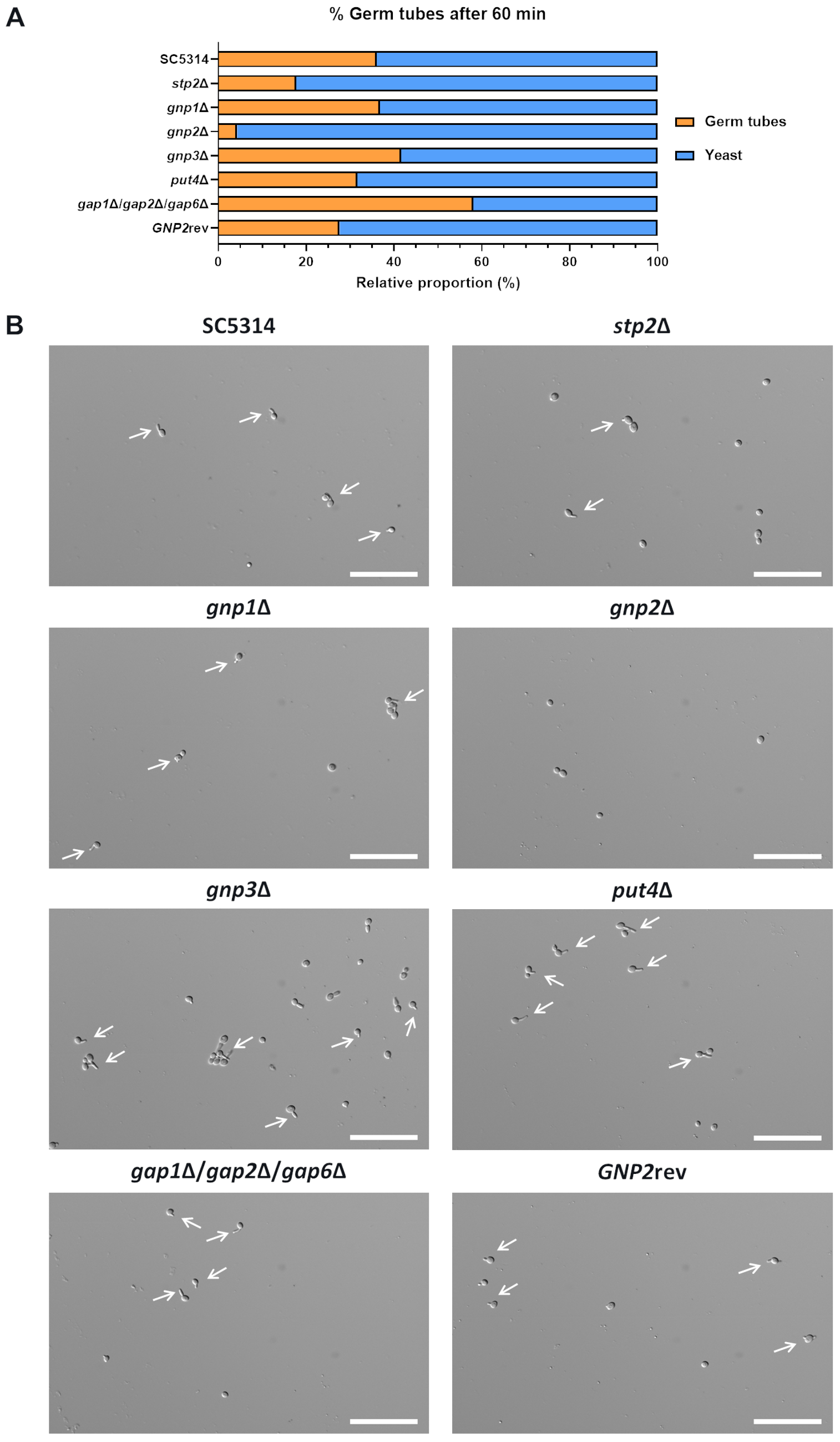

Supplement: FIG S6 [file mbio.03142-21-sf006.tif]

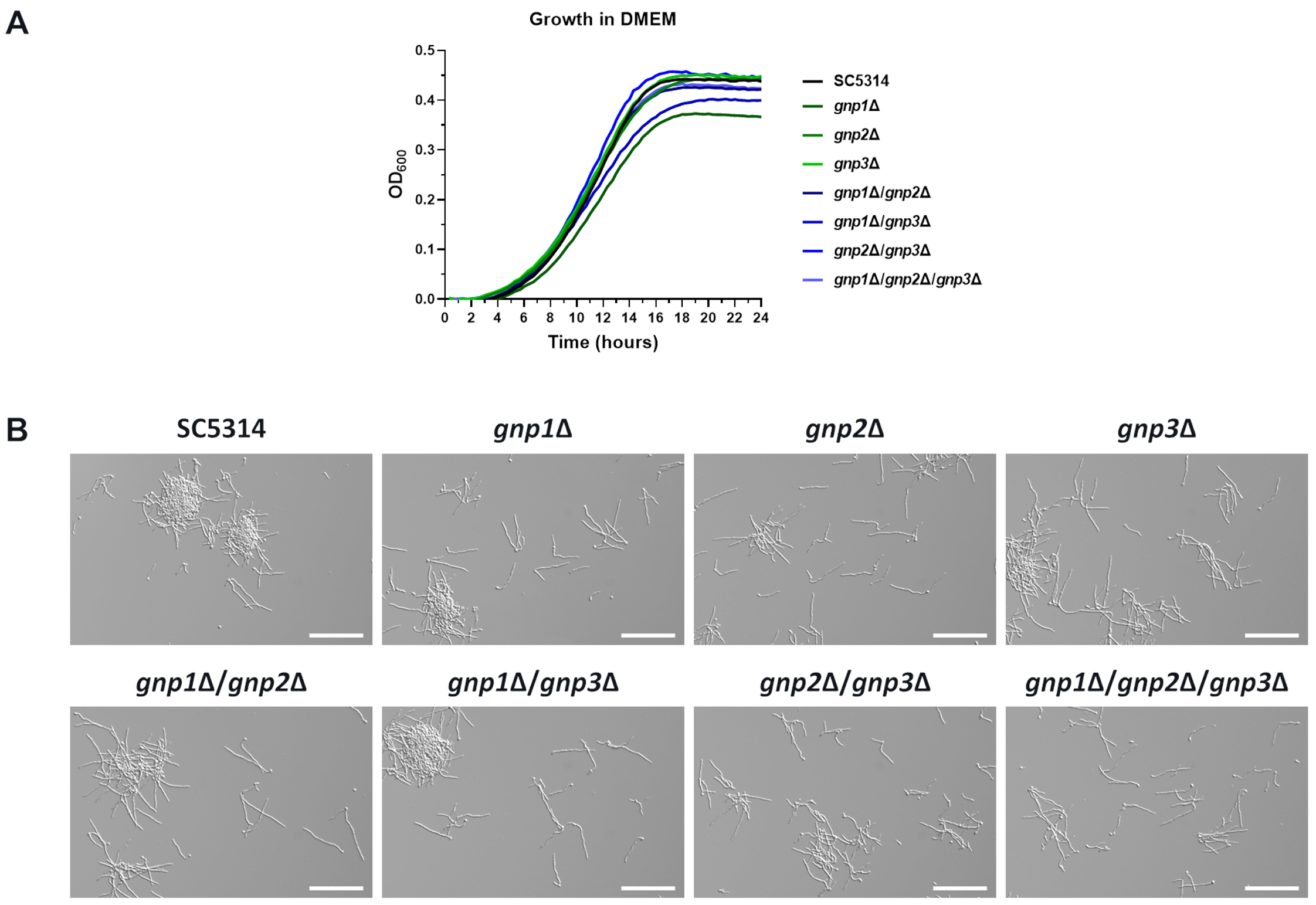

Supplement: FIG S7 [file mbio.03142-21-sf007.tif]
